# Association between Different Types of Plant-Based Diets and Risk of Dyslipidemia: A Prospective Cohort Study

**DOI:** 10.3390/nu13010220

**Published:** 2021-01-14

**Authors:** Kyueun Lee, Hyunju Kim, Casey M. Rebholz, Jihye Kim

**Affiliations:** 1 Department of Medical Nutrition, Graduate School of East-West Medical Science, Kyung Hee University, Yongin 17104, Korea; kyueun07@khu.ac.kr; 2 Department of Epidemiology, Johns Hopkins Bloomberg School of Public Health, Baltimore, MD 21205, USA; hkim25@jhu.edu (H.K.); crebhol1@jhu.edu (C.M.R.); 3 Welch Center for Prevention, Epidemiology and Clinical Research, Johns Hopkins University, Baltimore, MD 21205, USA

**Keywords:** plant-based diets, Asians, dyslipidemia, plant food quality, prospective study

## Abstract

We evaluated the associations among different types of plant-based diet indices, risk of dyslipidemia, and individual lipid disorders in Asian populations with different dietary patterns from Western populations. Participants included 4507 Korean adults aged ≥40 years without dyslipidemia and related chronic diseases at baseline (2001–2002). Dietary intakes were assessed using an average of validated food frequency questionnaires measured twice. We calculated three plant-based diet indices: overall plant-based diet index (PDI), healthful plant-based diet index (hPDI), and unhealthful plant-based diet index (uPDI). During a follow-up of 14 years, 2995 incident dyslipidemia cases occurred. Comparing the highest with lowest quintiles, the multivariable-adjusted hazard ratios (HRs) for incident dyslipidemia were 0.78 (95% CI, 0.69–0.88) for PDI, 0.63 (95% CI, 0.56–0.70) for hPDI, and 1.48 (95% CI, 1.30–1.69) for uPDI (*P*-trend < 0.0001 for all). Associations between PDI and individual lipid disorders differed by sex. The PDI was inversely associated with risk of developing hypertriglyceridemia in men and with risk of developing low high-density lipoprotein cholesterol in women. The hPDI was inversely associated with risk of all lipid disorders, whereas the uPDI was positively associated with individual lipid disorders. The quality of plant foods is important for prevention of dyslipidemia in a population that consumes diets high in plant foods.

## 1. Introduction

Dyslipidemia is a major risk factor of cardiovascular disease (CVD), the top cause of death in the USA and worldwide [1]. Dyslipidemia is a common public health problem because of its high prevalence in the general population [2]. Vegan or vegetarian diets has been suggested as an important dietary strategies for prevention of cardiometabolic diseases [3]. However, clinical trials of vegetarian diets have shown mixed results on improving lipid levels [4,5,6,7]. These studies have only explored the short-term effect of vegetarian diets on blood lipids and scarce data exist on how plant foods consumed for a longer period is associated with lipid disorders. Furthermore, previous studies focused on only the restriction of animal food consumption with little consideration given to the type of plant foods consumed, although some plant foods, such as refined carbohydrates or plant foods high in sugar and salt, have a distinct impact on metabolic health [3,6,7,8].

Recently, investigators have developed new indices that considered gradations of adherence to a predominantly plant-based diet [3]. Plant-based diet indices, such as overall plant-based diet index (PDI), healthful plant-based diet index (hPDI), and unhealthful plant-based diet index (uPDI) assess intakes of plant foods and animal foods, considering healthiness of plant foods [9,10,11]. Greater adherence to PDI and hPDI have been associated with lower risk of type 2 diabetes, CVD, and kidney disease, whereas greater adherence to uPDI has been associated with higher risk of these chronic diseases [11,12,13]. Considering the growing interest and evidence of plant-based diets, it is meaningful to assess the newly established plant-based diet indices and risk of dyslipidemia, which is strongly predictive of several metabolic diseases.

Further, limited evidence on plant-based diets and chronic diseases are available in Asian populations who have different dietary patterns from western populations [14,15]. Specifically, Korean populations have followed diets rich in plant foods for a long period of time. Plant-based diets may have different associations in the Korean populations compared with western populations, who consume mainly diets high in animal foods.

In this context, we aimed to evaluate the associations between different types of plant-based diet indices (PDI, hPDI, uPDI) and risk of dyslipidemia and individual lipid disorders using data from a community-based cohort of middle-aged and older Korean adults.

## 2. Materials and Methods

### 2.1. Study Cohort

Analyses were based on data from the population-based cohorts in the Korean Genome and Epidemiology Study (KoGES) [16], which were conducted to investigate the genetic and environmental etiology of common complex diseases in Koreans. The KoGES included 10,030 participants (40–69 years of age) enrolled from Ansan and Ansung cities near Seoul. Participants were recruited into the study between 2001 and 2002 (baseline) and returned for biennial follow-up visits until 2016. The follow-up rate of cohort was 92%. The Institutional Review Boards of the Korea Centers for Disease Control and Prevention and Kyung Hee University (KHGIRB-19-398) approved the study protocol, and participants provided written informed consent.

For dyslipidemia analysis, individuals with extraordinary energy intake (*n* = 410), had CVD or cancer at baseline (*n* = 304), individuals who had dyslipidemia at baseline (*n* = 4076), refused to participate in follow-up examinations (*n* = 470), had missing information (*n* = 263), were excluded. Our final sample was 4507. Similarly, for the analysis of individual lipid disorders, 6373 individuals for hypertriglyceridemia, 5963 individuals for high cholesterolemia, 7176 individuals for low high-density lipoprotein cholesterol (HDL-C), 6110 individuals for high low-density lipoprotein cholesterol (LDL-C) and 6816 individuals for high total cholesterol/HDL-C.

### 2.2. Assessment of Plant-Based Diet Index Score

Participants’ usual intake of foods and beverages was assessed with a validated 106-item semi-quantitative food frequency questionnaire (FFQ) [17]. Reproducibility and validity of the FFQs have been described in details elsewhere [17]. In the prospective study, the FFQ was administered at baseline and visit 3 (2005–2006). To incorporate the two dietary assessments into the estimate, we used the cumulative average of dietary intake at baseline and visit 3. When participants developed dyslipidemia before visit 3 or did not complete the questionnaire at visit 3, only dietary intakes from baseline were used [18]. Participants reported the frequency and the portion size of food consumption over the previous year. The FFQ had 9 options representing frequency of consumption, ranging from “almost never” to “3 times per day,” and 3 options for portion size (0.5 serving, 1 serving, and 2 servings) [19]. Nutrient intakes were calculated using a Korean food composition table [20].

For the calculation of the PDI, hPDI, and uPDI scores, we used the approaches outlined in previous studies [10]. Briefly, all food items were categorized into 17 food groups for the PDI, hPDI, and uPDI. Food groups were classified as healthy plant foods (i.e., whole grains, fruits, vegetables, nuts, legumes, tea and coffee), less healthy plant foods (i.e., refined grains, potatoes, sugar-sweetened beverages, sweets and desserts, salty foods), and animal foods (i.e., animal fat, dairy, eggs, fish, meat, miscellaneous animal foods). We considered salty foods (i.e., kimchi and pickled vegetables with salt or soy sauce) as less healthy plant foods due to their high sodium content and prior associations with dyslipidemia in the Korean population [21]. We did not include vegetable oil and fruit juices as separate food groups, because the FFQ did not include items on oil intake, and fruits and fruit juices were asked together.

After grouping foods, we adjusted for total energy intake, and ranked participants into quintiles. For the PDI score, all plant foods (healthy and less healthy plant foods) were positively scored. For instance, subjects in the highest quintile of fruits consumption received a score of 5, and those in the lowest quintile received a score of 1. For the hPDI, only healthy plant foods were positively scored. For the uPDI, only less healthy plant foods were positively scored. Animal foods were negatively scored in all plant-based diet indices. For instance, those in the highest quintile of egg consumption received a score of 1, and those in the lowest quintile received a score of 5. After adding up the scores across these categories for plant and animal foods, we divided the overall diet scores into quintiles for analyses.

### 2.3. Measurements

Participants self-reported medical history and medication use. On an annual basis, trained staff measured participants’ height and weight, and blood specimens were collected. The study procedures have been described in detail previously [22]. Blood samples were collected after at least 8 h fasting and plasma was separated for biochemical measurements. After centrifuge, they were transported to a central clinical laboratory and samples were stored at −80 °C until analyses were conducted. The concentrations of triglycerides, total cholesterol, and HDL-C were enzymatically measured with an AutoAnalyzer (ADVIA 1650, Bayer HealthCare, Tarrytown, NY, USA) using a standardized protocol. LDL-C concentration was estimated by using the Friedewald formula as follows: LDL-C = total cholesterol—(triglyceride/5 + HDL-C) [23]. For reproducibility, a sub-sample (*n* = 40) was duplicated and tested. All laboratory assessments showed very high reproducibility (Pearson’s correlation >0.99) [24].

### 2.4. Ascertainment of Dyslipidemia

Dyslipidemia was defined as having one of the following symptoms [25]: (1) hypertriglyceridemia as plasma triglyceride concentration ≥5.18 mmol/L; (2) hypercholesterolemia as plasma total cholesterol ≥6.2 mmol/L; (3) low HDL-C as plasma HDL-C concentration <1.0 mmol/L; (4) high LDL-C as LDL-C concentration ≥4.1 mmol/L; or (5) use of anti-dyslipidemia medication. In addition, we used high total cholesterol/HDL-C to analyze the association between plant-based diets and HDL-C. High ratio of total cholesterol to HDL-C was defined ≥5.

### 2.5. Assessment of Covariates

Data on demographic characteristics and lifestyle factors were examined at baseline using structured questionnaires. Body mass index (BMI in kg/m^2^) was calculated from measured height (cm) and weight (kg). Residential location was assessed as urban or rural. Educational level was categorized as ≤6, 7 to 12, and >12 years. Pack-years of cigarettes were calculated by multiplying the number of packs of cigarettes smoked per day and the number of years the person has smoked. Alcohol consumption was calculated by summing alcohol intake from different types of alcoholic drinks consumed within one year [26]. Physical activity was evaluated using the metabolic equivalent of task (MET)-hours per day for each participant by accounting for types and intensity of physical activity [27].

### 2.6. Statistical Analysis

Characteristics of participants at baseline are expressed as mean and standard deviation (SD) or median and range (continuous variables) or number and percentage (categorical variables). Comparisons of variables across quintiles of plant-based diet indices were conducted by either chi-square tests or generalized linear regressions, as appropriate.

Hazard ratios (HRs) and 95% confidence intervals (CIs) for risk of dyslipidemia according to quintiles of plant-based diet indices were calculated using multivariable Cox proportional hazards models. We adjusted for the same set of covariates in both studies. Model 1 adjusted for age and sex. Model 2 was additionally adjusted for residence area, BMI, education level, physical activity, pack-years of cigarettes, alcohol consumption, and total energy intake. Linear trends were tested by using the median score within each quintile. Then, using the same set of covariates, we modeled all plant-based diet indices as a continuous variable and calculated measures of association per 1 SD higher score. Next, we used restricted cubic splines with 4 knots at the 5th, 35th, 65th, and 95th percentile to examine the shape of the associations. Next, we examined if plant-based diets were associated with individual lipid disorders (hypertriglyceridemia, hypercholesterolemia, low HDL-C, and high LDL-C) in the fully adjusted models. This analysis was separated by sex because the response of individual lipids to diet may differ by sex [28]. For the analysis of individual lipid disorders, menopausal status was added as a covariate in women.

Person time was calculated as the time from baseline examination until the date of dyslipidemia event or censoring. Censoring was defined as those who did not return for a follow-up visit. It is possible that participants did not return for a follow-up visit due to death or moving, but these data were not available.

The proportional hazard assumption was confirmed graphically using log–log plots and statistically using Schoenfeld residuals [29]. We did not find a violation of the assumption. All data were analyzed using SAS software, version 9.4 (SAS Institute, Cary, NC, USA). *p* < 0.05 was considered statistically significant for two-sided tests.

## 3. Results

Those in the highest quintiles of PDI and hPDI were more likely to be older, women, had lower education level and pack-years of cigarettes, consume lower amounts of alcohol, and more physically active (Table 1). Those in the highest quintile of uPDI were more likely to be older, men, had lower education level, higher pack-years of cigarettes, consume higher amounts of alcohol, and more physically active.

Those in the highest quintiles of all plant-based diet indices had a higher consumption of carbohydrate, lower consumption of protein, fat, and cholesterol (Table S1). Those in the highest quintiles of PDI and hPDI generally consumed more fiber, vitamins, and micronutrients than those in the lowest quintiles. On the contrary, those in the highest quintile of uPDI had lower amount of nutrients.

During a total follow up of 29,313 person-years, 2995 (66.5%) participants developed dyslipidemia. Comparing the highest with the lowest quintile, the multivariable-adjusted HR of incident dyslipidemia were 0.78 (95% CI, 0.73–0.92) for PDI, 0.63 (95% CI, 0.56–0.70) for hPDI, and 1.48 (95% CI, 1.30–1.69) for uPDI (*P*-trend < 0.0001 for all) (Table 2). The strong association remained after adjustment for anti-dyslipidemia medication (HR (95% CI) in the highest quintile: 0.78 (0.69–0.88) for PDI, 0.62 (0.55–0.70) for hPDI, 1.45 (1.27–1.66) for uPDI).

**Figure 1:**
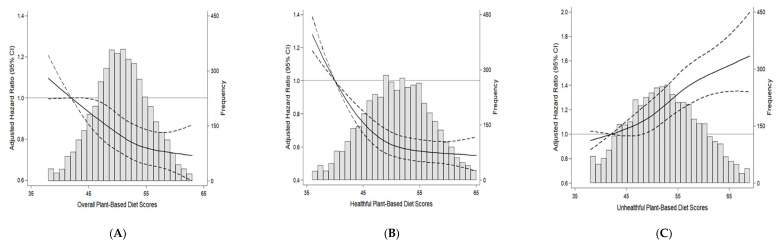
Multivariable adjusted hazard ratios and 95% confidence intervals for incident dyslipidemia according to the continuous plant-based diet index using restricted cubic splines. The histogram in gray shows the distribution of plant-based diet scores. The solid lines represent the multivariable adjusted hazard ratios for incident dyslipidemia, modeled using restricted cubic splines with 4 knots (5th, 35th, 65th, 95th percentiles). The reference was point was set at the 5th percentile of each score. The dashed lines represent 95% confidence intervals. (**A**) Overall plant-based diet score, (**B**) healthful plant-based diet score, (**C**) unhealthful plant-based diet score. Hazard ratios were adjusted for age (year, continuous), sex (men/women), residence area (rural/urban), education (≤6, 7–12, >12 years), physical activity (MET/day, continuous), pack-years of cigarettes (continuous), alcohol intake (g/day, continuous), body mass index (kg/m^2^, continuous), and total energy intake (kcal/day, continuous).

We found approximately linear associations between all plant-based diet indices and incident dyslipidemia when we examined the shape of associations (Figure 1). When plant-based diet indices were modeled continuously, 1 SD of PDI and hPDI was associated with 9% and 16% lower risk of incident dyslipidemia, respectively, and 1 SD of uPDI was associated with 16% higher risk of dyslipidemia after adjustment for confounders (*p* < 0.0001 for all).

Associations between plant-based diet indices and individual lipid disorders differed by sex (Table 3). Among men, PDI was inversely associated with risk of developing hypertriglyceridemia, hypercholesterolemia, and high LDL-C respectively. Among women, PDI was inversely associated with risk of developing hypercholesterolemia, low HDL-C, high LDL, and high total cholesterol/HDL-C. The hPDI was inversely associated with risk of all lipid disorders except for high total cholesterol/HDL-C in women whereas uPDI was positively associated with risk of all lipid disorders except for hypercholesterolemia in women.

## 4. Discussion

In a community-based cohort, greater adherence to PDI or hPDI was associated with a lower risk of incident dyslipidemia, whereas greater adherence to uPDI was associated with a higher risk, after adjusting for demographic characteristics and lifestyle factors.

Stronger inverse associations were observed for hPDI than PDI. Association between PDI and individual lipid disorders differed by sex. Among men, greater adherence to PDI was inversely associated with hypertriglyceridemia whereas among women, PDI was inversely associated with low HDL-C. Associations of hPDI or uPDI with risk of individual lipid disorders did not differ by sex. These findings highlight the importance of defining plant-based diets in terms of the quality of plant foods for the prevention and management of dyslipidemia. In addition, our results suggest sex differences for the association between plant-based diet and individual lipid disorders.

Our results were aligned with the findings of prior studies. A meta-analysis of clinical trials and cross-sectional studies reported that vegetarian diets were associated with lower levels of total cholesterol, LDL-C, and HDL-C [30]. Another feeding study demonstrated that low-carbohydrate plant-based diet for 4 weeks significantly reduced LDL-C and the ratio of total cholesterol to HDL-C over a high-carbohydrate and low-fat diet in overweight hyperlipidemic subjects [31].

A traditional Korean diet is comprised of a variety of grains and vegetables. Koreans consume various plant foods in every meal and their habitual diet has been similar to a plant-based diet for a long period of time. It has been shown that plant-based diets are low in saturated fat and cholesterol and high in unsaturated fat [3]. This lipid composition from plant-based diets leads to less absorption and conversion to blood cholesterol and reduce triglyceride concentration [32]. In addition, plant foods are rich in favorable compounds for preventing dyslipidemia, such as dietary fiber, phytosterols, antioxidants, and polyphenols. Dietary fiber increases cholesterol removal through binding bile acids and cholesterol [33]. Thus, low dietary fiber, and high carbohydrate from less healthy plant foods (e.g., refined grains and sweets and desserts), may increase triglyceride concentration. Soy protein in legumes may increase resistance to LDL-C oxidation and increase HDL-C [34]. Polyphenols improve lipid profiles by inhibiting the oxidation of LDL-C [35]. These beneficial components diets high in plant foods and low in animal foods can exert substantial influence on lipid profiles via multiple mechanisms.

Interestingly, hPDI was inversely associated with low HDL-C, whereas uPDI was positively associated with low HDL-C in contrast to the result from a meta-analysis showing a positive association between vegetarian diet (overall plant-based diet) and low HDL-C [30]. The difference between studies may be because previous studies did not consider the healthiness of plant foods and they only observed short-term effects of vegetarian diets on HDL-C. Our study suggests that habitual consumption of plant-based diets consumed for a long period of time may play an important role in raising HDL-C concentration. Moreover, the quality of plant foods should be considered to identify the impact of plant-based diet on HDL-C.

The association between PDI and individual lipid disorders differed by sex. An inverse association of PDI with hypertriglyceridemia or high LDL-C was observed in men whereas the association with low HDL-C was observed only in women. The mechanism for sex difference is not clear. Different metabolic responses to individual lipid disorders may be due to differences in dietary habits between men and women. Women have dietary behaviors more similar to plant-based diets than men [36,37], whereas men prefer animal foods, assuming that men would consume higher intake of saturated fat. Thus, diets rich in plant foods might have a stronger relationship with LDL-C in men who followed diets higher in animal foods [38]. On the other hand, women consume more white rice and noodles, fruits, and vegetables than men [39] and these dietary habits would result in high carbohydrate intake than fat, which were positively associated with low HDL-C among Koreans [40,41].

Our study evaluated prospective associations between different types of plant-based diets and risk of dyslipidemia using recently developed plant-based diet indices. Our results expand the understanding of how diets rich in plant foods for a longer period may be associated with dyslipidemia by influencing different types of lipids.

Strengths of our study include the use of data from a population-based cohort, validated FFQ, repeated dietary assessments, and sufficient follow-up period to ascertain incident dyslipidemia cases. Our data also contribute to the literature with a unique focus on Asian populations who have different dietary patterns than western populations. However, several limitations need to be taken into account. We made slight changes to the categorization of foods, because certain less healthy plant foods and healthy plant foods were asked together. Further, there was no data on vegetable oil intake in this population, which could have affected blood lipids. However, vegetable oil would be the predominant source of fat consumption for most participants. Lastly, although we adjusted for important confounders, there may still be residual confounding factors.

## 5. Conclusions

In populations who habitually consume diets rich in plant foods, great adherence to three types of plant-based diets were differentially associated with risk of incident dyslipidemia. Our study strongly supports considering the quality of plant foods for dyslipidemia prevention. Prospective studies are needed to confirm the relationship between a plant-based diet and dyslipidemia in diverse populations with different dietary habits.

## Figures and Tables

**Table 1:** Baseline characteristics of study participants according to quintiles of plant-based diet indices in the Korean adults.

		Q1	Q2	Q3	Q4	Q5
**Overall Plant-Based Diet Index**						
Sample size, *n*		886	934	1038	826	823
Median score (range)		44 (32–46)	48 (47–49)	51 (50–52)	54 (53–55)	58 (56–71)
Female, *n* (%)		488 (55.1)	535 (57.3)	609 (58.7)	490 (59.3)	519 (63.1)
Age, years		49.9 (8.6)	50.9 (8.9)	51.9 (9.1)	52.4 (8.7)	53.7 (9.1)
Residential location, *n* (%)						
	Rural, Ansung	400 (45.2)	436 (46.7)	515 (49.6)	425 (51.5)	482 (58.6)
	Urban, Ansan	486 (54.8)	498 (53.3)	523 (50.4)	401 (48.5)	341 (41.4)
Edu Education level, *n* (%)						
	≤6 years	215 (24.3)	283 (30.3)	333 (32.1)	292 (35.4)	350 (42.5)
	7–12 years	518 (58.5)	519 (55.6)	591 (56.9)	446 (54.0)	411 (49.9)
	>12 years	153 (17.2)	132 (14.1)	114 (11.0)	88 (10.6)	62 (7.6)
Pack-years of cigarettes, pack/year		7.9 ± 13.9	8.2 ± 15.9	7.8 ± 15.4	7.8 ± 14.9	8.1 ± 16.1
Alcohol intake, g/day		10.0 ± 21.0	9.7 ± 22.0	8.9 ± 19.7	7.4 ± 16.6	7.4 ± 20.8
Body Mass Index, kg/m^2^		23.9 ± 3.0	23.9 ± 3.0	24.0 ± 3.2	23.9 ± 3.3	24.3 ± 3.2
Physical activity, MET/day		21.7 ± 14.8	23.2 ± 15.0	23.6 ± 15.2	25.1 ± 15.6	26.2 ± 15.8
Total energy intake, kcal/day		2045 ± 696	1955 ± 636	1907 ± 597	1912 ± 588	1846 ± 589
Triglycerides, mmol/L		1.2 ± 0.4	1.2 ± 0.4	1.2 ± 0.4	1.2 ± 0.4	1.2 ± 0.4
LDL-C, mmol/L		3.0 ± 0.6	2.9 ± 0.7	3.0 ± 0.7	2.9 ± 0.7	2.9 ± 0.6
HDL-C, mmol/L		1.4 ± 0.3	1.4 ± 0.3	1.4 ± 0.3	1.4 ± 0.3	1.4 ± 0.2
Total cholesterol, mmol/L		4.9 ± 0.7	4.9 ± 0.7	4.9 ± 0.7	4.9 ± 0.7	4.9 ± 0.7
Healthy plant food, servings/day		9.4 ± 4.7	10.5 ± 4.7	10.9 ± 5.0	12.1 ± 4.9	12.8 ± 5.1
Less healthy plant food, servings/day		6.3 ± 2.7	6.7 ± 2.8	7.0 ± 2.9	7.4 ± 3.0	7.8 ± 3.1
Animal food, servings/day		3.9 ± 2.7	3.4 ± 1.9	3.1 ± 1.8	3.0 ± 1.7	2.4 ± 1.5
**Healthful plant-based diet index**						
Sample size, *n*		890	959	788	982	888
Median score (range)		43 (30~45)	48 (46~49)	51 (50~52)	54 (53~56)	59 (57~73)
Female, *n* (%)		409 (46.0)	507 (52.9)	457 (58.0)	622 (63.3)	888 (72.8)
Age, years		49.0 (8.3)	51.2 (8.9)	51.8 (9.0)	52.7 (9.0)	53.6 (9.0)
Residential location, *n* (%)						
	Rural, Ansung	293 (32.9)	458 (47.8)	426 (54.1)	557 (56.7)	524 (59.0)
	Urban, Ansan	597 (67.1)	501 (52.2)	362 (45.9)	425 (43.3)	364 (41.0)
Education level, *n* (%)						
	≤6 years	188 (21.1)	274 (28.6)	263 (33.4)	371 (37.8)	377 (42.5)
	7–12 years	543 (61.0)	562 (58.6)	428 (54.3)	505 (51.4)	447 (50.3)
	>12 years	159 (17.9)	123 (12.8)	97 (12.3)	106 (10.8)	64 (7.2)
Pack-years of cigarettes, pack/year		10.2 ± 16.2	8.8 ± 14.9	8.1 ± 15.9	7.0 ± 13.8	5.7 ± 15.2
Alcohol intake, g/day		11.6 ± 22.2	9.7 ± 20.7	8.7 ± 19.6	7.3 ± 17.3	6.6 ± 20.4
Body Mass Index, kg/m^2^		24.1 ± 3.0	23.8 ± 3.0	23.8 ± 3.1	24.1 ± 3.3	24.2 ± 3.3
Physical activity, MET/day		21.6 ± 14.3	23.4 ± 15.8	25.0 ± 15.0	24.6 ± 15.6	25.0 ± 15.5
Total energy intake, kcal/day		1929 ± 607	2007 ± 647	1952 ± 630	1888 ± 609	1894 ± 629
Triglycerides, mmol/L		1.2 ± 0.4	1.2 ± 0.4	1.2 ± 0.4	1.2 ± 0.4	1.2 ± 0.4
LDL-C, mmol/L		3.0 ± 0.6	3.0 ± 0.6	2.9 ± 0.7	2.9 ± 0.7	2.9 ± 0.7
HDL-C, mmol/L		1.4 ± 0.2	1.4 ± 0.3	1.4 ± 0.3	1.4 ± 0.3	1.4 ± 0.3
Total cholesterol, mmol/L		5.0 ± 0.7	4.9 ± 0.7	4.9 ± 0.7	4.9 ± 0.7	4.8 ± 0.7
Healthy plant food, servings/day		9.2 ± 4.2	10.9 ± 5.4	11.1 ± 5.0	11.5 ± 4.9	12.6 ± 5.0
Less healthy plant food, servings/day		8.4 ± 3.0	7.8 ± 2.8	7.2 ± 2.9	6.3 ± 2.5	5.4 ± 2.5
Animal food, servings/day		3.8 ± 1.9	3.7 ± 2.1	3.3 ± 2.1	2.8 ± 1.6	2.3 ± 1.5
**Unhealthful plant-based diet index**						
Sample size, *n*		902	885	995	955	770
Median score (range)		44 (30–46)	49 (47–50)	52 (51–54)	57 (55–59)	62 (60–75)
Female, *n* (%)		642 (71.2)	535 (60.5)	565 (56.8)	485 (50.8)	414 (53.8)
Age, years		48.7 (7.8)	50.1 (8.4)	51.8 (8.9)	52.7 (9.2)	55.4 (9.1)
Residential location, *n* (%)						
	Rural, Ansung	248 (27.5)	339 (38.3)	488 (49.1)	588 (61.6)	595 (77.3)
	Urban, Ansan	654 (72.5)	546 (61.7)	507 (50.9)	367 (38.4)	175 (22.7)
Education level, *n* (%)						
	≤6 years	132 (14.6)	223 (25.2)	331 (33.3)	387 (40.5)	400 (52.0)
	7–12 years	595 (66.0)	529 (59.8)	547 (55.0)	483 (50.6)	331 (43.0)
	>12 years	175 (19.4)	133 (15.0)	117 (11.7)	85 (8.9)	39 (5.0)
Pack-years of cigarettes, pack/year		5.0 ± 12.6	7.6 ± 14.4	8.0 ± 15.2	9.7 ± 16.6	9.6 ± 16.8
Alcohol intake, g/day		6.7 ± 16.3	8.9 ± 19.5	9.4 ± 21.3	10.2 ± 23.3	8.4 ± 19.3
Body Mass Index, kg/m^2^		24.1 ± 3.1	24.2 ± 3.2	24.2 ± 3.1	23.9 ± 3.1	23.8 ± 3.3
Physical activity, MET/day		20.6 ± 12.7	22.9 ± 14.2	23.9 ± 16.1	25.8 ± 16.1	26.8 ± 16.8
Total energy intake, kcal/day		2075 ± 583	2054 ± 633	1931 ± 648	1875 ± 626	1707 ± 556
Triglycerides, mmol/L		1.1 ± 0.4	1.2 ± 0.4	1.2 ± 0.4	1.2 ± 0.4	1.3 ± 0.4
LDL-C, mmol/L		2.9 ± 0.6	3.0 ± 0.6	3.0 ± 0.6	2.9 ± 0.7	2.9 ± 0.7
HDL-C, mmol/L		1.4 ± 0.3	1.4 ± 0.3	1.4 ± 0.3	1.4 ± 0.3	1.4 ± 0.3
Total cholesterol, mmol/L		4.9 ± 0.7	4.9 ± 0.7	4.9 ± 0.7	4.9 ± 0.7	4.8 ± 0.7
Healthy plant food, servings/day		14.7 ± 5.0	12.8 ± 4.7	11.1 ± 4.3	9.3 ± 3.7	7.0 ± 3.5
Less healthy plant food, servings/day		5.9 ± 2.5	6.6 ± 2.8	7.1 ± 3.0	7.5 ± 3.0	8.0 ± 3.0
Animal food, servings/day		4.5 ± 2.1	3.8 ± 1.9	3.2 ± 1.6	2.5 ± 1.5	1.7 ± 1.2

Data are expressed as mean± SD or *n* (%). Abbreviations: HDL-C, high density lipoprotein-cholesterol; LDL-C, low density lipoprotein-cholesterol; MET, Metabolic equivalent of task.

**Table 2:** Hazard ratios and 95% confidence intervals for incident dyslipidemia according to quintiles of plant-based diet scores among Korean adults.

	Overall Plant-Based Diet Index					Healthful Plant-Based Diet Index					Unhealthful Plant-Based Diet Index				
	Q1	Q2	Q3	Q4	Q5	Q1	Q2	Q3	Q4	Q5	Q1	Q2	Q3	Q4	Q5
**No of participants (no of cases)**	886 (602)	934 (635)	1038 (668)	826 (538)	823 (552)	890 (617)	959 (639)	788 (516)	982 (646)	888 (577)	902 (560)	885 (559)	995 (657)	955 (672)	770 (547)
**Person years**	5392	5798	7059	5489	5576	4628	6204	5371	6843	6268	6673	6168	6351	5894	4228
**Model 1 ^a^**	1.00	0.95 (0.85–1.06)	0.82 (0.73–0.91)	0.83 (0.74–0.94)	0.82 (0.73–0.93)	1.00	0.75 (0.67–0.84)	0.68 (0.61–0.77)	0.66 (0.59–0.74)	0.63 (0.56–0.71)	1.00	1.07 (0.95–1.21)	1.19 (1.06–1.33)	1.30 (1.16–1.45)	1.43 (1.27–1.62)
**P-trend**	<0.0001					<0.0001					<0.0001				
**Model 2 ^b^**	1.00	0.94 (0.84–1.05)	0.80 (0.72–0.90)	0.82 (0.73–0.92)	0.78 (0.69–0.88)	1.00	0.75 (0.67–0.84)	0.69 (0.61–0.78)	0.65 (0.58–0.72)	0.63 (0.56–0.70)	1.00	1.07 (0.95–1.20)	1.19 (1.06–1.34)	1.33 (1.18–1.50)	1.48 (1.30–1.69)
**P-trend**	<0.0001					<0.0001					<0.0001				
**P-interaction for sex**	0.8170					0.2439					0.1897				
**Per SD ^c^**	0.91 (0.87–0.94)					0.84 (0.81–0.88)					1.16 (1.11–1.21)				

Q, quintile. ^a^ Model 1 was adjusted for age (year, continuous) and sex (men/women). ^b^ Model 2 was additionally adjusted for residence area (rural/urban), education (≤6, 7–12, >12 years), physical activity (MET/day, continuous), pack-years of cigarettes (continuous), alcohol intake (g/day, continuous), body mass index (kg/m^2^, continuous), and total energy intake (kcal/day, continuous). ^c^ In the continuous analysis, hazard ratios expressed per 1 standard deviation (SD). The PDI had a SD of 5.2, hPDI had a SD of 6.4, and uPDI had a SD of 7.0.

**Table 3:** Hazard ratios (HRs) and 95% confidence intervals (CIs) for individual lipid disorders according to quintiles of plant-based diet scores among Korean adults.

	Overall Plant-Based Diet Index					Healthful Plant-Based Diet Index					Unhealthful Plant-Based Diet Index				
	Q1	Q2	Q3	Q4	Q5	Q1	Q2	Q3	Q4	Q5	Q1	Q2	Q3	Q4	Q5
**Hypertriglyceridemia**															
**Men**															
**No of participants (no of cases)**	608 (202)	526 (163)	646 (193)	515 (135)	508 (129)	590 (220)	599 (177)	521 (143)	577 (147)	516 (135)	600 (164)	538 (162)	611 (176)	528 (167)	526 (153)
**Model ^a^**	1.00	0.93 (0.76–1.14)	0.90 (0.74–1.09)	0.81 (0.65–1.01)	0.75 (0.60–0.94)	1.00	0.69 (0.56–0.84)	0.62 (0.50–0.76)	0.58 (0.47–0.71)	0.61 (0.49–0.76)	1.00	1.24 (0.99–1.54)	1.17 (0.94–1.46)	1.44 (1.14–1.80)	1.33 (1.04–1.69)
**P-trend**	0.0056					<0.0001					0.0092				
**Women**															
**No of participants (no of cases)**	703 (171)	733 (167)	833 (207)	638 (157)	663 (180)	704 (190)	736 (178)	651 (154)	771 (181)	708 (179)	772 (159)	686 (153)	756 (188)	605 (162)	748 (220)
**Model**	1.00	0.88 (0.71–1.08)	0.91 (0.74–1.11)	0.90 (0.72–1.12)	0.92 (0.74–1.14)	1.00	0.81 (0.66–0.99)	0.76 (0.62–0.95)	0.69 (0.56–0.85)	0.76 (0.62–0.93)	1.00	1.14 (0.91–1.42)	1.28 (1.03–1.60)	1.34 (1.07–1.69)	1.60 (1.27–2.01)
**P-trend**	0.6063					0.0037					<0.0001				
**Hypercholesterolemia**															
**Men**															
**No of participants (no of cases)**	686 (111)	653 (78)	736 (77)	621 (80)	626 (64)	666 (105)	740 (92)	815 (75)	669 (77)	632 (61)	716 (90)	629 (75)	723 (94)	630 (73)	624 (78)
**Model**	1.00	0.73 (0.55–0.98)	0.63 (0.47–0.84)	0.82 (0.61–1.09)	0.67 (0.49–0.91)	1.00	0.75 (0.56–0.99)	0.74 (0.55–1.00)	0.68 (0.50–0.91)	0.58 (0.42–0.80)	1.00	1.05 (0.77–1.43)	1.27 (0.95–1.71)	1.30 (0.94–1.79)	1.62 (1.15–2.27)
**P-trend**	0.0319					0.0011					0.0036				
**Women**															
**No of participants (no of cases)**	516 (130)	552 (145)	604 (116)	487 (106)	482 (95)	505 (136)	561 (132)	496 (115)	578 (121)	501 (88)	571 (128)	499 (120)	578 (130)	452 (108)	541 (106)
**Model**	1.00	1.01 (0.80–1.28)	0.71 (0.55–0.91)	0.81 (0.63–1.05)	0.71 (0.54–0.93)	1.00	0.79 (0.62–1.00)	0.82 (0.64–1.05)	0.66 (0.52–0.85)	0.54 (0.41–0.71)	1.00	1.25 (0.97–1.60)	1.20 (0.94–1.55)	1.36 (1.04–1.78)	1.28 (0.96–1.71)
**P-trend**	0.0028					<0.0001					0.0582				
**Low HDL-C**															
**Men**															
**No of participants (no of cases)**	552 (303)	528 (276)	626 (336)	482 (251)	493 (277)	553 (316)	560 (298)	501 (263)	567 (279)	500 (287)	562 (280)	525 (275)	591 (305)	484 (277)	519 (306)
**Model**	1.00	0.93 (0.79–1.09)	0.90 (0.77–1.05)	0.85 (0.72–1.01)	0.86 (0.72–1.01)	1.00	0.74 (0.63–0.87)	0.67 (0.57–0.80)	0.57 (0.48–0.67)	0.70 (0.59–0.82)	1.00	1.09 (0.92–1.29)	1.09 (0.92–1.29)	1.24 (1.04–1.48)	1.32 (1.10–1.59)
**P-trend**	0.0365					<0.0001					0.0014				
**Women**															
**No of participants (no of cases)**	669 (303)	719 (332)	810 (359)	594 (280)	637 (282)	676 (305)	705 (322)	613 (282)	737 (324)	698 (323)	710 (249)	646 (257)	749 (340)	611 (344)	713 (376)
**Model**	1.00	0.95 (0.81–1.10)	0.84 (0.72–0.98)	0.87 (0.74–1.03)	0.76 (0.64–0.89)	1.00	0.88 (0.75–1.03)	0.80 (0.68–0.95)	0.71 (0.60–0.83)	0.73 (0.62–0.86)	1.00	1.18 (0.99–1.40)	1.37 (1.15–1.62)	1.68 (1.41–2.00)	1.72 (1.44–2.05)
**P-trend**	0.0006					<0.0001					<0.0001				
**High LDL-C**															
**Men**															
**No of participants (no of cases)**	698 (126)	674 (86)	757 (100)	647 (97)	644 (68)	696 (134)	732 (111)	638 (84)	703 (87)	651 (61)	737 (105)	649 (101)	751 (95)	636 (83)	647 (93)
**Model**	1.00	0.70 (0.53–0.92)	0.69 (0.53–0.89)	0.84 (0.64–1.10)	0.58 (0.43–0.78)	1.00	0.75 (0.58–0.97)	0.63 (0.48–0.83)	0.57 (0.43–0.74)	0.43 (0.32–0.59)	1.00	1.20 (0.91–1.58)	1.10 (0.83–1.46)	1.29 (0.95–1.74)	1.69 (1.24–2.32)
**P-trend**	0.0059					<0.0001					0.0034				
**Women**															
**No of participants (no of cases)**	755 (224)	771 (198)	858 (220)	649 (169)	723 (186)	722 (221)	798 (235)	665 (168)	814 (194)	757 (179)	791 (205)	724 (217)	826 (221)	642 (168)	773 (186)
**Model**	1.00	0.81 (0.67–0.99)	0.78 (0.65–0.94)	0.81 (0.66–0.99)	0.79 (0.65–0.97)	1.00	0.88 (0.73–1.06)	0.76 (0.62–0.93)	0.67 (0.55–0.81)	0.66 (0.54–0.81)	1.00	1.30 (1.07–1.57)	1.21 (1.00–1.47)	1.26 (1.01–1.56)	1.28 (1.02–1.59)
**P-trend**	0.0360					<0.0001					0.0656				
**High total cholesterol/HDL-C**															
**Men**															
**No of participants (no of cases)**	514 (213)	534 (189)	627 (243)	530 (196)	517 (198)	513 (246)	574 (218)	547 (168)	578 (214)	510 (193)	510 (186)	546 (202)	508 (197)	600 (245)	558 (209)
**Model**	1.00	0.84 (0.69–1.02)	0.94 (0.78–1.13)	0.88 (0.72–1.07)	1.01 (0.83–1.23)	1.00	0.69 (0.58–0.83)	0.53 (0.43–0.64)	0.60 (0.50–0.73)	0.65 (0.54–0.79)	1.00	1.02 (0.84–1.25)	1.18 (0.96–1.45)	1.32 (1.08–1.61)	1.26 (1.01–1.56)
**P-trend**	0.8018					<0.0001					0.0035				
**Women**															
**No of participants (no of cases)**	675 (228)	714 (255)	787 (291)	617 (274)	633 (283)	668 (236)	720 (283)	620 (251)	718 (264)	700 (297)	612 (196)	603 (185)	727 (275)	749 (322)	735 (353)
**Model**	1.00	1.02 (0.85–1.22)	1.02 (0.86–1.22)	1.30 (1.09–1.55)	1.21 (1.01–1.45)	1.00	1.09 (0.91–1.29)	1.05 (0.88–1.26)	0.87 (0.73–1.04)	1.01 (0.85–1.20)	1.00	1.02 (0.84–1.25)	1.33 (1.10–1.61)	1.60 (1.32–1.93)	2.12 (1.74–2.58)
**P-trend**	0.0020					0.2770					<0.0001				

Q, quintile. ^a^ Model was adjusted for age (year, continuous), residence area (rural/urban), education (≤6, 7–12, >12 years), physical activity (MET/day, continuous), pack-years of cigarettes (continuous), alcohol intake (g/day, continuous), body mass index (kg/m^2^, continuous), total energy intake (kcal/day, continuous), and menopausal status (yes/no, only for women).

## Data Availability

Data underlying the results of our study are not publicly available due to KoGES data policy. Data are available from the Division of Genetic Epidemiology and Health Index, NIH, Korea Centers for Disease Control and Prevention (contact via Mi-Jin Cho at whalwls0227@korea.kr) for researchers who meet the criteria for access to confidential data.

## Supplementary Materials

The following are available online at https://www.mdpi.com/2072-6643/13/1/220/s1, Table S1: Nutritional characteristics of diet according to quintiles of plant-based diet indices among Korean adults.
