# Supplementary material for: Association between Different Types of Plant-Based Diets and Risk of Dyslipidemia: A Prospective Cohort Study
Source: Nutrients. 2021 Jan 14;13(1):220. doi: 10.3390/nu13010220 (PMC7828805; doi:10.3390/nu13010220)
Supplement: Supplementary file 1 [file nutrients-13-00220-s001.pdf]

Supplemental information

Table S1. Nutritional characteristics of diet according to quintiles of plant-based diet indices among Korean adults.

	Quintile 1	Quintile 2	Quintile 3	Quintile 4	Quintile 5	*P*-value
			**Overall plant-based diet index**			
Total energy intake, kcal/day	2045±696	1955± 636	1907± 597	1912±588	1846±589	<0.0001
Carbohydrate, % of energy	69.3 (7.9)	71.0 (7.6)	72.6 (6.9)	72.7 (6.5)	74.5 (5.9)	<0.0001
Protein, % of energy	14.0 (2.4)	13.8 (2.5)	13.4 (2.3)	13.5 (2.3)	13.1 (2.0)	<0.0001
Fat, % of energy	16.7 (5.8)	15.2 (5.6)	14.0 (5.1)	13.9 (4.8)	12.4 (4.3)	<0.0001
Calcium, mg/1000 kcal	239.9±99.2	240.6±97.3	237.8±95.8	251.9±101.0	248.6±95.1	0.0078
Phosphorus, mg/1000 kcal	519.6±87.2	520.5±91.4	518.8±89.0	531.9±92.3	530.0±84.3	0.0017
Iron, mg/1000 kcal	5.1±1.4	5.4±1.4	5.5±1.4	5.8±1.4	6.0±1.5	<0.0001
Potassium, mg/1000 kcal	1154±332	1251±357	1282±349	1377±363	1457±367	<0.0001
Niacin, mg/1000 kcal	7.9±1.7	7.9±1.7	7.8±1.5	8.0±1.5	8.1±1.5	0.0029
Vitamin C, mg/1000 kcal	52.8±34.3	62.3±35.9	63.6±34.5	72.0±36.7	78.0±38.1	<0.0001
Zinc, mg/1000 kcal	4.6±1.2	4.5±1.3	4.4±1.1	4.4±1.1	4.3±1.0	<0.0001
Vitamin B-6, mg/1000 kcal	0.9±0.2	0.9±0.2	0.9±0.2	1.0±0.2	1.0±0.2	<0.0001
Folate, µg /1000 kcal	106.5±36.4	120.3±43.3	124.7±40.2	136.1±43.9	146.9±48.6	<0.0001
Beta-carotene, µg/1000 kcal	1113±764	1284±883	1354±950	1481±945	1653±1122	<0.0001
Fiber, g/1000 kcal	2.9±1.0	3.4±1.1	3.6±1.1	4.0±1.2	4.4±1.3	<0.0001
Vitamin E, mg/1000 kcal	4.4±1.5	4.6±1.5	4.6±1.4	4.8±1.4	5.0±1.5	<0.0001
Cholesterol, mg/1000 kcal	107.9±54.9	94.6±54.0	83.9±51.9	78.9±47.5	63.8±42.2	<0.0001
			**Healthful plant-based diet index**			
Total energy intake, kcal/day	1929±607	2007± 647	1952±630	1888±609	1894±629	<0.0001
Carbohydrate, % of energy	69.0 (6.9)	70.7 (7.2)	72.3 (7.2)	73.3 (6.8)	74.7 (6.7)	<0.0001
Protein, % of energy	14.0 (2.3)	13.8 (2.5)	13.5 (2.4)	13.4 (2.2)	13.2 (2.2)	<0.0001
Fat, % of energy	16.9 (5.1)	15.5 (5.2)	14.3 (5.2)	13.4 (5.0)	12.2 (4.9)	<0.0001
Calcium, mg/1000 kcal	256.2±91.7	251.4±94.8	241.5±95.3	237.6±102.3	229.8±101.6	<0.0001
Phosphorus, mg/1000 kcal	522.3±82.9	523.3±89.8	519.0±88.8	522.9±92.1	530.9±90.7	0.0822
Iron, mg/1000 kcal	5.3±1.3	5.5±1.4	5.5±1.5	5.6±1.5	5.9±1.5	<0.0001
Potassium, mg/1000 kcal	1264±319	1308±362	1293±361	1294±377	1339±409	0.0006
Niacin, mg/1000 kcal	8.2±1.6	8.1±1.7	7.9±1.5	7.8±1.5	7.7±1.6	<0.0001
Vitamin C, mg/1000 kcal	57.3±26.3	65.4±33.9	67.2±39.4	66.7±38.5	70.4±42.7	<0.0001
Zinc, mg/1000 kcal	4.6±1.3	4.5±1.3	4.4±1.3	4.4±0.9	4.3±0.9	0.0003

Vitamin B-6, mg/1000 kcal	0.9±0.2	0.9±0.2	0.9±0.2	0.9±0.2	0.9±0.2	0.2222
Folate, µg /1000 kcal	120.4±41.1	126.0±42.7	124.6±42.3	127.4±46.5	133.1±48.5	<0.0001
Beta-carotene, µg/1000 kcal	1358±847	1430±923	1378±963	1355±976	1325±1046	0.1799
Fiber, g/1000 kcal	3.2±1.0	3.5±1.1	3.6±1.2	3.8±1.2	4.1±1.3	<0.0001
Vitamin E, mg/1000 kcal	4.8±1.3	4.8±1.5	4.7±1.5	4.5±1.4	4.6±1.6	<0.0001
Cholesterol, mg/1000 kcal	108.1±49.3	96.0±53.6	85.2±52.5	78.5±49.1	63.3±47.0	<0.0001
**Unhealthful plant-based diet index**						
Total energy intake, kcal/day	2075±583	2054± 633	1931±648	1875±626	1707±557	<0.0001
Carbohydrate, % of energy	67.4 (6.4)	69.5 (6.3)	71.4 (6.7)	74.3 (6.0)	78.1 (5.9)	<0.0001
Protein, % of energy	15.2 (2.1)	14.4 (2.0)	13.8 (2.1)	12.7 (1.8)	11.5 (1.7)	<0.0001
Fat, % of energy	17.5 (4.8)	16.1 (4.8)	14.9 (5.1)	12.9 (4.6)	10.4 (4.6)	<0.0001
Calcium, mg/1000 kcal	299.4±98.0	266.5±89.7	247.4±91.5	214.7±87.7	181.5±77.1	<0.0001
Phosphorus, mg/1000 kcal	590.2±81.3	553.5±73.3	530.6±79.0	489.7±70.9	445.2±65.9	<0.0001
Iron, mg/1000 kcal	6.5±1.3	5.9±1.3	5.7±1.4	5.1±1.2	4.4±1.2	<0.0001
Potassium, mg/1000 kcal	1488±347	1369± 326	1326±358	1201±335	1087±345	<0.0001
Niacin, mg/1000 kcal	9.0±1.5	8.4±1.5	8.1±1.5	7.5±1.3	6.8±1.3	<0.0001
Vitamin C, mg/1000 kcal	75.6±39.9	68.0±37.4	65.7±35.9	60.7±33.4	55.7±34.1	<0.0001
Zinc, mg/1000 kcal	4.9±1.3	4.6±0.9	4.5±1.3	4.2±1.1	3.9±0.7	<0.0001
Vitamin B-6, mg/1000 kcal	1.0±0.2	1.0±0.2	0.9±0.2	0.9±0.2	0.8±0.2	<0.0001
Folate, µg /1000 kcal	141.6±41.9	131.9±41.1	130.5±47.5	117.5±42.0	107.7±41.8	<0.0001
Beta-carotene, µg/1000 kcal	1574±1060	1445±936	1404±941	1239±874	1163±887	<0.0001
Fiber, g/1000 kcal	3.9±1.1	3.7±1.2	3.7±1.3	3.5±1.2	3.4±1.2	<0.0001
Vitamin E, mg/1000 kcal	5.5±1.4	5.1±1.4	4.7±1.3	4.2±1.2	3.8±1.4	<0.0001
Cholesterol, mg/1000 kcal	119.5±50.5	101.4±48.5	89.8±51.5	68.5±40.6	47.4±39.2	<0.0001
